# Editorial Department

**Published:** 1856-04

**Authors:** 


We shall be glad to receive and forward subscriptions to the work described below. It will be published by Messrs. Lippincott, Grambo & Co., in their usual excellent style. Knowing, as we do, Mr. Blodget's qualifications for a work so important to the medical man, we hope it may have a large circulation in the profession.

*Climatology of the United States, and of the Temperate Latitudes of the North American Continent.* Embracing a full comparison of these, with the Climatology of the Temperate Latitudes of Europe and Asia. And especially in regard to Agriculture, Sanitary Investigations, and Engineering, with Isothermal and Rain Charts, for each Season, the Extreme Months, and the Year. Including a Summary of the Statistics of Meteorological Observations in the United States, condensed from recent Scientific and Official Publications. By LORIN BLODGET, Author of several recent Reports on American Climatology, Member of the National Institute, and of various Learned Societies. One volume large octavo, 450 pages. Price Four Dollars.

*Prospectus.*—The above work, constituting a thorough treatise upon the Climatology of the Temperate Latitudes of North America, and particularly of the new Interior and Pacific Districts of the United States, is now nearly ready for the press, and will be published as soon as a sufficient number of subscriptions are received to form a basis for the first edition.

It will be illustrated by twelve superior engravings, on a new Outline Map 10 by 22 inches, embracing the whole North American Continent from the twenty-fifth to the fiftieth degree of north latitude. Five of these are Illustrations of the Distribution of Heat in Isothermal lines, lithographed in colors, and five are Shaded and Tinted Charts, representing the Distribution of Rain. The former are after the model of Humboldt's Isothermal Lines; the latter are new—both being new as positive scientific results for the North American Continent. The next is an illustration of the Comparative Temperatures of the two Continents in Temperate Latitudes, similar to Humboldt's Isothermal Chart, but embracing the corrections derived from the most recent observations, those in the United States coming down to June, 1855. All these results are based on the most complete summaries of nearly all existing American observations, including those taken by the Officers of the Medical Department of the United States Army.

The volume also embraces the Report on the Distribution of Temperature and of Rain in the United States, based on and accompanying the Climatological Statistics of the Surgeon General's Office, recently prepared for publication by the Author.

